# Surgical Treatment of Sprengel’s Deformity: A Systematic Review and Meta-Analysis

**DOI:** 10.3390/children8121142

**Published:** 2021-12-06

**Authors:** Paola Zarantonello, Giovanni Luigi Di Gennaro, Marco Todisco, Piergiorgio Cataldi, Stefano Stallone, Andrea Evangelista, Daniele Ferrari, Diego Antonioli, Giovanni Trisolino

**Affiliations:** 1 Department of Pediatrics Orthopedics and Traumatology, IRCCS Istituto Ortopedico Rizzoli, 40136 Bologna, Italy; giovanniluigi.digennaro@ior.it (G.L.D.G.); todiscomarco.97@gmail.com (M.T.); piergiorgio.cataldi@ior.it (P.C.); stallone.stefano@gmail.com (S.S.); daniele.ferrari@ior.it (D.F.); diego.antonioli@ior.it (D.A.); giovanni.trisolino@ior.it (G.T.); 2 Unit of General Affairs, IRCCS Istituto Ortopedico Rizzoli, 40136 Bologna, Italy; andrea.evangelista@ior.it

**Keywords:** Sprengel, rare disease, child, shoulder, scapula, congenital, deformity, surgery, Green, Woodward

## Abstract

(1) Background: Sprengel’s deformity (SD) is a rare congenital anomaly caused by failure in the descent of the scapula. We aimed to systematically review the current literature reporting data from children undergoing surgery for SD, in order to explore the rate of success and complications of the different surgical techniques, possibly providing recommendations about the management of SD in children. (2) Methods: we electronically searched the literature from Ovid, MEDLINE and the Cochrane Library databases. Demographic data, surgical procedures, outcomes and complications were analyzed. We categorized surgical procedures into five groups. (3) Results: 41 articles met the inclusion criteria, showing a poor overall study quality; 674 patients (711 shoulders) were analyzed. Green’s and Woodward’s procedures, both aiming the scapular relocation in a more anatomical position, were the most commonly used techniques. We counted 168 adverse events (18 major complications). The best clinical and cosmetic results seem to be achieved when surgery is performed in children aged less than eight years. (4) Conclusions: this paper represents the first systematic review reporting qualitative and quantitative data about the surgical treatment of SD. Surgery for SD seems to be effective in increasing the shoulder’s range of motion and improving the cosmetic appearance in almost all cases, with a low rate of major complications.

## 1. Introduction

The congenital elevation of the scapula, or Sprengel’s deformity (SD), is a rare congenital deformity of the shoulder girdle, characterized by failure in the descent of the scapula to the normal position during development, leading to a hypoplastic, elevated and malrotated scapula [1,2]. It represents the most common congenital abnormality of the shoulder in children and it can be found in association with other disorders such as Klippel–Feil syndrome and scoliosis [1,3,4]. It shows a female predominance, mostly a unilateral presentation, and is more frequently found on the left side.

SD was first identified by Eulemberg in 1863 [4]; subsequently, Willet and Walsham [5,6] reported two cases, giving a detailed anatomical description of the deformity and identifying a potential association with spine and rib anomalies. In 1891, Sprengel [7] described four cases and the condition became associated with his name [8].

The disorder varies in severity from a slightly limited shoulder range of motion (ROM) and mild cosmetic deformity to more pronounced dysfunction and severe clinical abnormalities [1]. The Cavendish classification is commonly used for clinical assessment and, according to this, SD is classified into four grades—grade 1: shoulders are level, very mild deformity is observed, almost invisible when covered by clothes; grade 2: the deformity is still mild, appearing as a bump, but glenohumeral joints are level; grade 3: moderate deformity with 2–5 cm of visible elevation of the affected shoulder; grade 4: severe deformity with >5 cm elevation of the affected shoulder, accompanied by neck webbing. A modified classification is rarely used and adds a grade 0 in cases in which the shoulders are at the same level and there is no abnormality of the superior aspect of scapula [9]. The radiographic classification, developed by Rigault et al. [10], is based on the projecton of the superomedial angle of the scapula in relation with the associated vertebral level. According to this classification, in grade 1 the superomedial scapula angle is below T1, in grade 2 it lies between T1 and C5 and in grade 3 it is above C5.

An accurate and premature diagnosis is essential, and surgery is indicated in severe cases. Several surgical techniques have been described in the literature with satisfactory outcomes generally reported. The main techniques currently used for the treatment of SD can be distinguished into five groups [11,12]:

(1) Release of the medial muscle attachments, lowering the scapula and securing the inferior pole to the rib or the surrounding muscles. The main procedures belonging to this group are the Putti or Putti–Schrock procedure and Green’s procedure.
  a. Putti’s procedure, which is uncommon and obsolete, involves the lowering of the scapula and its fixing to the rib. Schrock’s modified procedure adds the osteotomy of the supraspinous scapular region and the acromial base to facilitate scapular descent [13].
  b. Green’s procedure, via a midline incision, consists of extraperiosteal detachment of the medial and superior scapular muscles, excision of the supraspinous portion, lowered positioning of the scapula and reattachment of the muscles. In the modified technique, the inferior angle of the scapula ias placed into a pocket created beneath the latissimus dorsi muscle, eliminating the need for a traction system, previously secured by a percutaneous wire [14,15]. Green’s procedure provides a good visualization of the supero-medial scapular angle, better access to the acromiothoracic vasculo-nervous pedicle and an intraoperative exposition of the scapula, which allows one to achieve a good scapular mobilization. It is considered a difficult technique, frequently associated with a wide scar-keloid.
(2) Release of the trapezius, elevator scapulae and rhomboids at the origins from the spinous processes, placing the scapula in an inferiorly-located pocket and reattaching the muscles inferiorly. The most widespread technique was reported by Woodward [16,17] in an attempt to simplify surgery and decrease complications. Woodward’s procedure seems the most physiological and the best biomechanically adapted, since it displaces more distally the origin of muscle insertion. On the other hand, it does not modify the muscular insertion and the scapular rotation and its complexity is increased in cases of spinal deformity, such as scoliosis or vertebral abnormalities.
(3) Mears’s procedure. Through a transverse incision centered on the scapular spine, this procedure consists of the muscular detachment at the scapular origin and the excision of the superomedial angle. It adds an oblique osteotomy through the body of the scapula to avoid the impingement of the residual portions of the bone during full abduction. The literature has reported the need for the resection of at least 50% of the body to obtain an adequate ROM. The technique includes a release of the long head of the triceps, which is considered by the author as a passive limitation of the abduction [18,19].
(4) Partial scapulectomy, consisting in the excision of the superomedial scapular angle. This procedure is performed, separately or in association with other techniques, when this portion is redundant [20,21]. This treatment does not improve scapular function, but it has a role in cases with mild disfigurement.
(5) Vertical scapular osteotomy (VSO). Following a vertical paramedial incision, the scapula is osteotomized about 1 cm from its vertebral border, the supero-medial part of the scapula is freed of all muscle attachments and the major lateral portion is lowered and reattached. This category includes the König–Wittek procedure [22], which was rapidly abandoned, because of scar-keloids, difficulties in osteotomy and postoperative bone irregularities, as well as poor functional improvement [23].

Currently, there are no systematic reviews reporting cumulative data on the efficacy of the different techniques. Therefore, we aimed to systematically review the articles reporting the surgical treatment of SD, to assess the quality of the studies, the reported outcomes and complications and possibly to provide further evidence and recommendations concerning the surgical treatment of SD.

## 2. Materials and Methods

### 2.1. Study Design

The review process was conducted according to the Preferred Reporting Items for Systematic Reviews and Meta-Analyses (PRISMA) guidelines [24].

### 2.2. Search Strategy

An electronic literature search of the Ovid, MEDLINE and Cochrane Library databases was conducted in March 2021 by two observers (P.C. and M.T.) using the terms “Sprengel”, “Sprengel deformity”, “Sprengel shoulder”, “congenital high scapula”, “omovertebral bone”, “Woodward procedure/technique” and “Green procedure/technique”. The search was then replicated using the appropriate MeSH terms. We did not limit our search by year of publication, kind of journal or level of evidence. All bibliographies were checked for further relevant studies.

### 2.3. Eligibility Criteria

Two authors reviewed all abstracts for potential relevance according to the following inclusion criteria: (1) original articles about Sprengel deformity, (2) written in English, (3) reporting surgical management, (4) performed in children (<18 years of age at the time of surgery), (5) involving three or more cases, (6) peer reviewed. The following exclusion criteria were applied: (1) studies not reporting original research, including review articles, expert opinion or current concepts articles; (2) posters or abstracts at annual meetings or masters’ theses without subsequent peer-reviewed publication of an article; (3) animal studies; (4) articles not written in English; (5) studies not reporting surgical management; (6) studies reporting only adult cases (>18 years); (7) case reports or studies reporting less than 3 cases. Nonetheless, case reports (<3 cases) were screened in order to highlight possible rare events and major complications, thus balancing the potential overrepresentation of positive results of our review.

### 2.4. Article Selection

Two non-blinded authors (P.C., M.T.) reviewed the titles and abstracts of each article identified in the literature search. If a study met all the criteria or the abstract did not provide enough information to include or exclude the report, full texts were obtained, reviewed and considered for data extraction. Whenever an agreement about study inclusion could not be resolved by consensus between the two reviewers, a third author (P.Z.) decided about the inclusion.

### 2.5. Data Extraction, Analysis and Critical Appraisal

Data extraction was conducted independently by 2 authors (P.C. and M.T.) on articles retrieved through the systematic search and classified as relevant for the pooled or the meta-analysis. The authors separately collected data from full texts manually, compiling an Excel spreadsheet (Microsoft, Redmond, Washington, DC, USA), whereas a third author (P.Z.) checked the accuracy and agreement of the extracted data to minimize subjective evaluation.

The following items were collected from each study and inserted into a predefined table: first author, publication year, demographics (patient’s sex, affected side, age at surgery), associated anomalies, presence and type of connection of the affected scapula, preoperative and postoperative assessments (shoulder‘s ROM, Cavendish’s and Rigault’s classifications), aspects related to the surgical treatment (kind of treatment, kind and length of immobilization, type of rehabilitation and length of follow-up), complications and outcomes.

Since many articles reported original techniques with minimal modifications of the main surgical procedures, we distinguished the main type of treatment into five groups [11,12]: group 1: Putti–Schrock’s and Green’s procedures; group 2: Woodward’s or modified Woodward’s procedures; group 3: Mears’s technique; group 4: VSO; group 5: partial scapular resection.

Complications were classified according to the Clavien–Dindo-Sink (CDS) classification, stratifying on the basis of the need for additional medical and/or surgical treatment [25,26]. We considered as minor complications such conditions that have no or minimal clinical relevance, requiring no other medical treatments or causing little deviation from the normal postoperative course (grade 1 or 2), i.e., unsightly scar, keloid, superficial infection and transient nerve palsy. In contrast, major complications require surgical or radiological unplanned intervention (grade 3 of the CDS classification), such as deep infections, persistent nerve palsy, hardware breakage or recurrence of the deformity. Life-threatening conditions are classified as grade 4 of the CDS classification, whereas grade 5 corresponds to the death of the patient.

Clinical outcomes were differentiated in clinician-derived and patient-derived assessments, specifying the applied score. In particular, post-operative evaluations using Cavendish’s scoring system, ROM assessment and/or Rigault’s classification were considered clinician-derived outcomes, whereas patient-reported scores, such as the Disabilities of the Arm, Shoulder and Hand (DASH) outcome questionnaire [27], the Simple Shoulder Test (SST) score [28] and the Pediatric Outcomes Data Collection Instrument (PODCI) [29], were considered patient-derived outcomes.

### 2.6. Statistical Analysis

The quality of the studies retrieved in the systematic review was assessed according to the Oxford Center for Evidence-Based Medicine (CEBM), the Newcastle–Ottawa Scale (NOS) and the Modified Coleman Methodology Score (mCMS) [30,31]. Non-parametric statistical methods were used to assess correlations and associations, based on the nature of variables. Studies reporting raw data regarding pre-operative and post-operative variables of interest were further investigated and included in the meta-analysis. We reported results analyzing Cavendish’s classification, the Rigault system, the final ROM and the rate of complications. The pooled incidence of successful outcomes, in terms of an improvement in Cavendish and/or Rigault scores, was reported as a percentage along with a 95% confidence interval (CI). To simplify the statistical analysis, we grouped cases with a score of 0 in the modified Cavendish’s classification with those with a score of 1 in the original system. Moreover, patients who reached 0 or 1 in Cavendish’s grading system, starting from grades 3 or 4, were considered as complete clinical success; patients who improved by at least one grade in the Cavendish score were considered as partial improvement cases. Random-effect models were used to provide a pooled estimate. Between-study heterogeneity was assessed with the Cochrane Q statistic and I^2^ tests. The source of heterogeneity was explored via subgroup analyses, according to type of procedure and age at intervention (≤8, >8 years) [12,32,33,34]. Potential publication bias was evaluated by the visual inspection of funnel plot asymmetry and Egger’s weighted regression tests. Statistical analyses were performed by using STATA 11.2 and R 4.1.1.

## 3. Results

### 3.1. Studies Included and Quality Assessment

**Figure 1:**
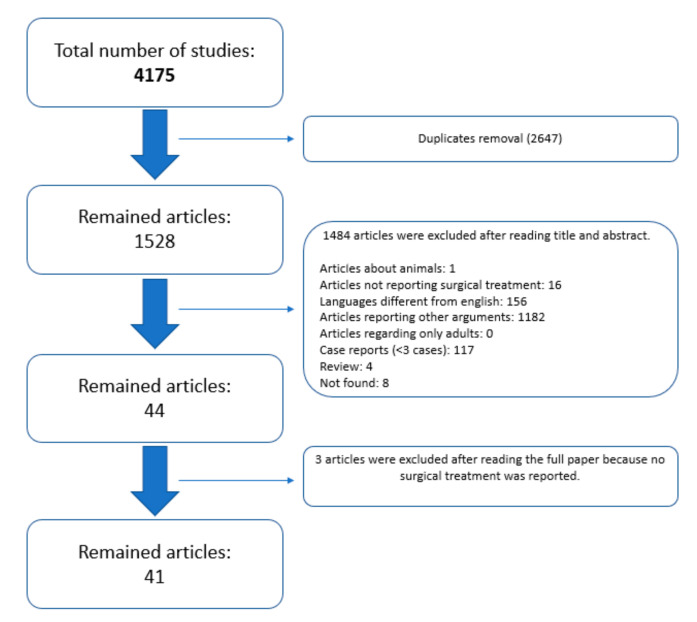
Flowchart highlighting the study acquisition details.

The search strategy yielded 4175 returns from three databases and reference lists. The removal of duplicates left 1528 articles to be screened for inclusion. Following title and abstract screening, 44 articles were identified for full text review, with 41 included in the final review (Figure 1).

All studies were case series, which were considered level IV of CEBM. The mean Newcastle–Ottawa and modified Coleman scores, of 6 points and 52.7 points, respectively, indicated inherent systematic deficiencies in the studies due to the poor overall study quality (Table 1). Moreover, the quality of the studies did not significatively change across years (NOS: Spearman rho 0.26; *p* = 0.098; mCMS: Spearman’s rho 0.31; *p* = 0.051) with only a weak improvement of the study reporting.

Most studies reported the patients’ sex, except three [34,43,57], and the affected side, except eight [21,23,43,47,50,54,57,59]. In the preoperative evaluation, 28 studies classified patients according to the Cavendish clinical scoring system [2,9,14,16,17,19,20,21,23,33,35,36,37,38,41,42,44,45,46,47,48,49,52,53,55,58,59,60] and 15 studies [14,15,16,17,19,21,32,36,41,45,48,53,57,58,60] applied Rigault’s radiographic classification. Ten papers [2,13,18,34,39,40,43,51,54,56] reported neither of them, whereas Khairouni et al. [50] described a personal score that analyzed the position of the scapula based on the vertebral level. The mentioned studies reported the same scores as well in the postoperative assessment, except Bhasker [41], who did not report the postoperative Rigault classification. Only two papers used patient-reported postoperative scores for clinical and functional assessment, such as the DASH Score [58], the SST Score [16,58] and the PODCI score [16], whereas most studies reported only subjective evaluations.

### 3.2. Demographic Data

The collected data are summarized in Supplementary Table S1.

A total of 674 patients, 37 of whom were bilateral (711 shoulders), were evaluated for this review. Considering those papers that reported patients’ sex and affected sides, we observed a slight predominance of the female sex (364/630; 57.8%) and the left side (282/514 54.9%). The mean age at the time of treatment was 5.9 years (range, 1–17 years). Klippel–Feil (KF) syndrome was found in 151 patients (22.4%) and scoliosis (SC) in 186 cases (27.6%); spina bifida (SB) was present in 54 cases (8%), whereas different rib anomalies were found in 105 patients (15.6%). Twenty-four patients (3.6%) presented renal abnormalities, especially the presence of a unilateral kidney (17 cases, 2.5%), whereas 16 cases (2.4%) showed appendicular anomalies, mainly foot and finger deformities (such as syndactyly, hypoplastic finger and clubfoot).

### 3.3. Clinical and Radiological Presentation

In the preoperative evaluation, all but three studies [43,51,53] reported the ROM of the shoulder in terms of abduction and/or elevation. All patients showed clinical deformity of the shoulder and reduced ROM. The mean preoperative maximum abduction angle was 104.07° (range: 81–127.69°), whereas the mean maximum elevation angle was 106.19° (range: 83–143.33°). No other symptoms were reported, apart from Greitemann et al. [23] who described arm and cervicothoracic pain in seven patients and a chronic headache in one case.

Twenty-three studies (441 patients) reported the grade of deformity, according to the Cavendish classification [9,14,16,17,19,20,21,23,35,36,37,41,42,44,45,46,48,52,53,55,58,59,60]. Ninety-seven patients (22%) were grade 4, 283 patients (64.2%) were grade 3, 51 patients (11.5%) were grade 2 and 10 patients (2.3%) were grade 1. Fifteen studies (283 patients) used Rigault’s classification [14,15,16,17,19,21,32,36,41,45,48,53,57,58,60]: 41% reported score 3 (116/283), 59% score 2 (167/283), whereas no patient had score 1 at the preoperative assessment. Only two papers did not report any vertebral-shoulder connection in their patients [9,40]; the other studies identified a connection between the scapula and spine in 305 patients (46.9%), represented by an omovertebral bone bar (264 patients), fibrous bridge (22 cases) or cartilaginous bar (19 cases).

### 3.4. Surgery

Surgical procedures were well-described in all studies. The collected data are shown in Supplementary Table S2.

The Putti–Schrock procedure was reported only by Farsetti [32] and Sulamaa [13] in nine cases. The original and modified Green procedure was reported in 14 papers [14,15,23,34,35,37,39,40,44,45,47,48,51,53], accounting for 280 patients; Woodward’s procedure (or modified techniques) was performed in 18 studies [2,9,16,17,23,32,33,42,43,46,49,50,54,55,56,57,58,60], accounting for 234 patients. Bhasker [41], Masquijo [19] and Mears [18] treated 29 patients overall with Mears’s technique. Sixty-three patients were treated with VSO [36,38,52,59]; only Greitemann [23] reported 10 patients treated with the König–Wittek procedure [22], whereas 38 patients were treated with a simpler partial scapulectomy [20,21,23,32]. Farsetti et al. [32] and Greitemann et al. [23] treated their cases with mixed procedures. All authors, except Khairouni [50] and Klisić [51], reported the period of follow-up in their papers. The mean follow-up was 6.25 years (range: 12 months–28 years). All studies, except for seven of them [16,23,33,37,42,57,61], reported the length and type of immobilization. In the studies reviewed, the mean period of immobilization was 3 weeks (range, 0.29–6 weeks), usually with a Velpeau or similar bandages.

### 3.5. Postoperative Assessment

All studies except three [43,51,53] reported the post-operative ROM of the shoulder in terms of abduction and/or elevation, with a maximum mean abduction angle of 147.74° (range: 97–180°) and a mean elevation angle of 148.78° (range: 105–175°). Overall, all patients showed an improvement in the shoulder’s ROM (Supplementary Table S2).

Twenty-three studies (383 patients) reported the postoperative Cavendish grade [2,9,14,16,17,19,20,21,33,35,36,37,41,42,44,45,46,48,52,55,58,59,60]. Of them, 16 studies (236 patients) [14,17,19,20,21,35,36,37,41,44,46,52,55,58,59,60] reported preoperative and postoperative raw data. Fourteen studies (219 patients) reported the postoperative Rigault classification [14,15,16,17,19,21,32,36,41,45,48,57,58,60]; of these, eight studies [14,15,17,21,32,36,58,60] reported preoperative and post-operative radiological raw data. A total of 18 studies (236 patients) reported preoperative and postoperative raw data for the Cavendish score (10 studies), Rigault score (two studies) or both (six studies), thus allowing for meta-analysis. Characteristics of patients are reported in Table 2.

**Figure 2:**
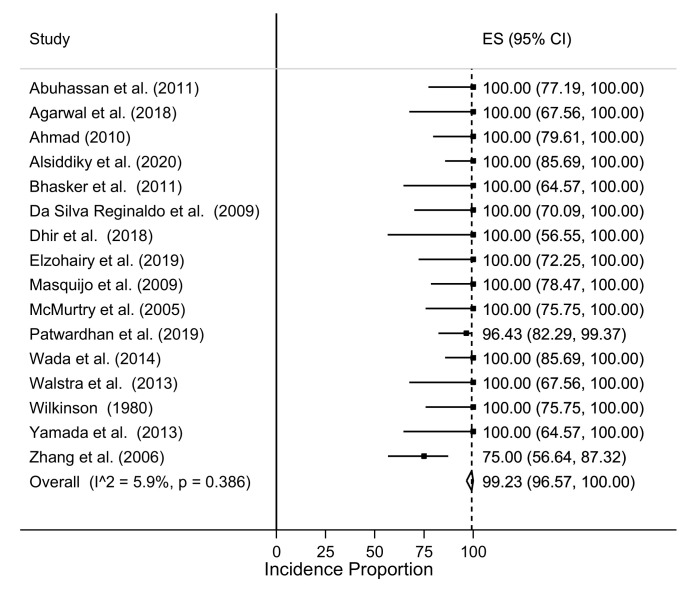
Incidence of Cavendish score improvement (at least one point) for single studies.

The results in terms of postoperative Cavendish grades are showed in Supplementary Table S2. An overall improvement of at least one point was reported in 99.2% of cases (95% CI: 96.6–100%), with no significant heterogeneity among studies (I^2^ = 5.9% *p* = 0.386), as seen in Figure 2.

**Figure 3:**
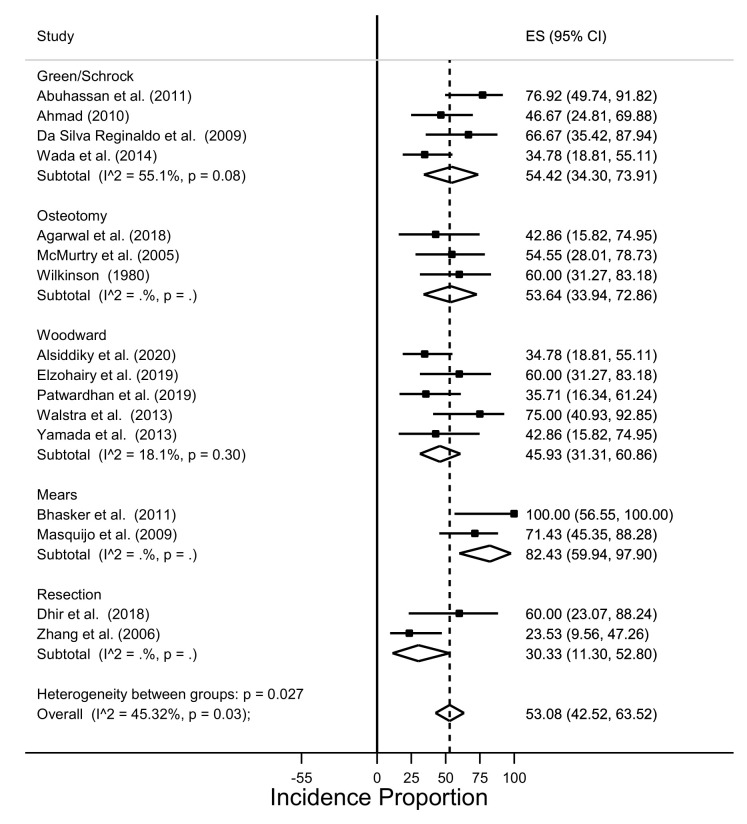
Incidence of postoperative Cavendish score 1 in cases with Cavendish score 3/4 at baseline.

Egger’s test did not suggest significant asymmetry in the funnel plot (*p* = 0.071). Nonetheless, the clinical improvement from a baseline grade of 3–4 to a postoperative grade of 1–0 was observed only in 53.1% of cases (95% CI: 42.5–63.5%) with significant heterogeneity between studies (I^2^ = 45.3% *p* =0.03). The surgical technique was significantly correlated with the outcome, with the best results observed for Mears’s procedure (82.4%) and worst results for the resection (30.3%), although subgroup analysis showed significant heterogeneity between groups (*p* = 0.03) (Figure 3).

In particular, the severity of the deformity at presentation was a significant factor, with children undergoing Green’s and Woodward’s procedures having a high frequency of severe patterns at baseline (*p* = 0.0005, Table 3).

**Figure 4:**
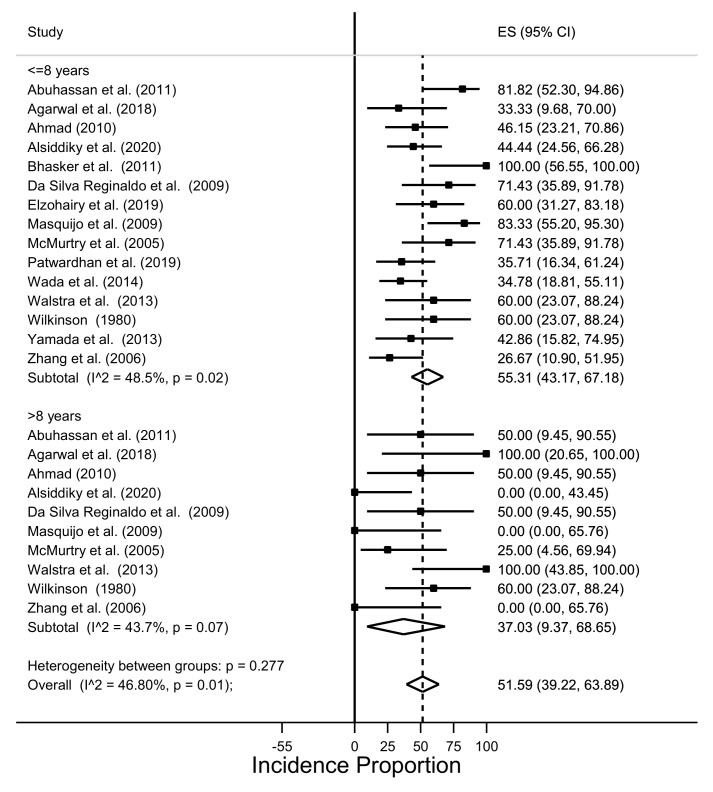
Incidence of postoperative Cavendish score 1 in cases with Cavendish score 3/4 at baseline based on the age at treatment.

In Figure 4, we investigated the effect of age at surgery on surgical correction, by grouping patients in two categories, considering the age of 8 years as a cut-off. Indeed, complete correction was reported in 55.3% (95% CI: 43.2–67.2%) of children aged 8 years or less, and in 37% (95% CI: 9.4–68.7%) in children older than 8 years. However, this correlation showed moderate heterogeneity (I^2^ = 46.8% *p* = 0.001).

**Figure 5:**
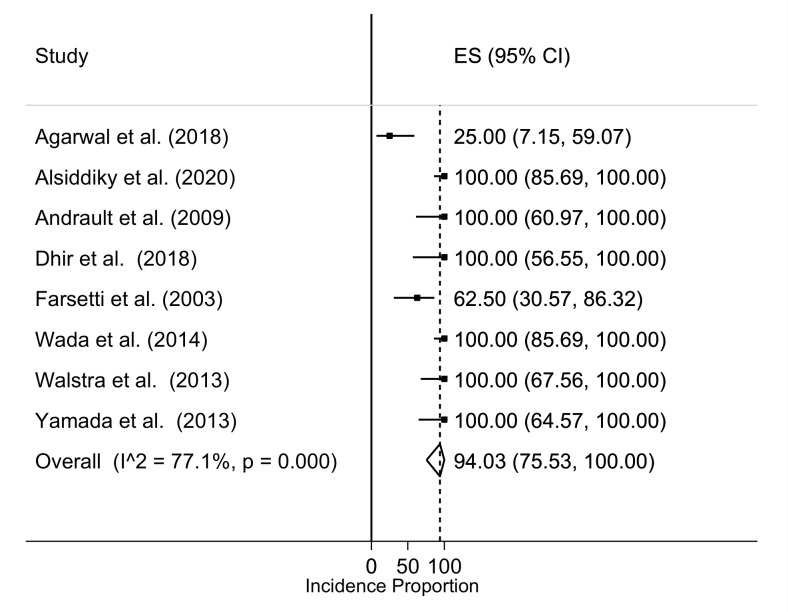
Incidence of Rigault score improvement (at least 1 point).

According to the Rigault classification, 54.8% of the studies received a score of 1 (120/219), 38.4% received a score of 2 (84/219), whereas 15 patients (6.8%) remained at a score of 3. The comparative analysis of the radiological success (defined as the improvement of at least 1 point in Rigault’s classification) demonstrated radiographic improvement in 94% of cases (95% CI 75.5–100%): However, the articles included in meta-analysis showed high heterogeneity (I^2^ = 77.1% *p* = 0.0001) (Figure 5).

Patient-derived outcome measures [27,28,29] were reported only in two studies [16,58], which applied the DASH Score [58], the SST Score [16,58] and the PODCI score [16], respectively. Walstra et al. [58] reported a postoperative DASH score of 14.59 and an SST score of 9.5, similar to the score reported by Ashok [16] (9.6), who also reported a postoperative PODCI score of 24.07.

### 3.6. Complications

Complications are reported in Supplementary Table S2. All papers, except six [17,21,36,49,54,56], reported at least one complication. We counted a total of 168 complications. Neither life-threatening complications (grade 4) nor the death of the patient (grade 5) were detected in the reviewed studies. One hundred and fifty cases had minor conditions, commonly related to wound problems such as as keloid, hypertrofic or unsightly surgical scars. Nerve palsies were reported in 11 cases (1.5%), and these were usually transient and did not require further hospitalization, and were thus categorized as grade 2 according to the CDS classification. We counted 18 major complications (2.5%), classified as grade 3 according to the CDS system. Among them, we noted a seroma that required surgical drainage [35]; a granuloma of the suture [16]; hardware removal due to breakage in two patients [44]; deep infection in two patients [51]; heterotopic ossifications causing limitations and pain in three patients [18,19,35]. Naik [53] described two patients with skin necrosis, one of whom needed a second intervention to resolve the problem; furthermore, he reported brachial plexus palsy and the loss of radial pulsation in one patient, requiring clavicle osteotomy, with recovery after 4 weeks. Lastly, pleural lesions occurred in three cases [51,53,58]. Three patients required revision surgery for incomplete correction or recurrence of the deformity [32,45,57].

We also screened 117 case reports (<3 cases). Among them, we found 20 articles, reporting surgical data of 20 children with SD, counting two major complications (10%) [62,63]: an atlantoaxial rotatory subluxation after surgical relocation of SD in a 5-year-old girl and the development of an extraabdominal desmoid tumor in the scar in a 8-year-old girl. The first patient was surgically treated to reduce the subluxation (reduction under general anesthesia and release of the subcutaneous tight band at the anterior part of trapezius muscle). The second case surgically underwent the total excision of the mass.

## 4. Discussion

This paper represents the first systematic review of the surgical treatment of SD, reporting qualitative and quantitative data, in order to provide evidence concerning the best treatment option in SD.

Although SD represents the most common congenital abnormality of the shoulder, it is a rare defect, with a reported incidence of 0.3 cases per 10,000 live births [64,65] and about 1000 cases described in literature. The disorder shows huge variety in terms of severity, in some cases causing considerable functional limitations and cosmetic problems [1,2]. SD is also associated with other congenital abnormalities, such as Klippel–Feil syndrome, congenital scoliosis, renal abnormalities and spina bifida in a considerable percentage of cases, thus impeding accurate physical examination upon presentation. Although mild SD generally does not require any treatment, moderate-to-severe SD usually requires surgery to improve aesthetics and increase shoulder motion. To the best of our knowledge, there are no studies reporting the efficacy of nonoperative treatments (brace, rehabilitation) for SD. Cosmetic appearance, according to Cavendish’s classification, and ROM are the most used indicators for treatment, although there is no well-established indication and timing for surgery. Vuillermin et al. [57] reported outcomes from 50 patients who were left untreated for SD. He described a gradual decline in the abduction range, with 26% of patients developing pain in adolescence.

Currently, there is no evidence regarding the best surgical option in SD. The significant heterogeneity among studies investigating different procedures did not allow comparisons among techniques. Our review showed that the most popular surgical techniques for SD were Green’s procedure and Woodward’s procedure, which were overall used in more than 70% of cases. Both Green’s and Woodward’s procedures, along with the Mears procedure, aim at relocating the scapula into a more anatomical position, providing better cosmetic aspects and increasing ROM. These procedures are currently preferred over partial scapulectomy and osteotomy.

In general, we can confirm that surgery for SD allows an increase in the shoulder’s ROM of about 40° in abduction and elevation and improves the cosmetic appearance of the posterior border of the neck in almost all cases, regardless of the technique used. The rate of major complications was 2.5%, thus showing that the surgical management of SD is safe and effective.

Nonetheless, a complete restoration of the position of the scapula, with a normal or nearly normal appearance of the posterior aspect of the neck (Cavendish’s grade 0–1), was achievable only in half of the cases. This aspect should be kept in mind when surgery for correcting SD is proposed to the child and their parents. Another important issue is that surgery seems to provide the best clinical and cosmetic results when performed in children aged less than eight years. We found that children with severe SD (Cavendish’s grade 3–4) and an age below eight years had complete or nearly complete restoration of the scapula position in approximately 55% of cases, compared to children aged more than eight years, who achieved complete or nearly complete correction in only 35% of cases. The reason could be that the muscolo-skeletal system of younger children may be more elastic, and it may have a greater ability to adapt to changes, thus allowing for greater correction and remodeling. Based on our findings, we suggest the early correction of SD, possibly below the age of eight.

Concerning the quality of the studies, we selected 41 studies among the 1528 potentially eligible papers, for a total of 674 patients that were surgically treated for SD. We categorized surgery into five groups. All the selected studies were retrospective case series, with most of them reporting merged data, thus not allowing for meta-analysis. Only 23 studies reported post-operative clinical outcomes according to the Cavendish classification and only 14 studies reported post-operative radiographic assessment according to the Rigault classification. Overall, the meta-analysis included a total of only 18 studies.

The lack of patient-derived outcome scores in almost all reports could not provide information concerning symptoms, pain, function, the quality of life of patients or the possibility of returning to daily life and sport activities. For assessing outcomes, most reports used the Cavendish classification, a largely qualitative classification assessed with the patient fully clothed and not accounting for ROM, as an indicator for surgery. Although many radiological measurements for SD have also been described, Rigault’s classification was the most frequently used, although its relevance is unclear as it is not known how well it correlates with clinical function or natural history [57].

Limitations of this review include the low level of evidence of all the included studies; the absence of comparative analysis, either prospective or retrospective; the lack of objective preoperative and postoperative scores and the limited use of clinical and radiological classifications, which makes this review prone to selection and observer bias. Although three literature databases were used, we might have missed possible relevant publications that were not listed in these libraries. The exclusion of non-English written reports further reduces the completeness of the information in our systematic review. Additionally, many studies had a small sample size, a lack of raw data and incomplete information about baseline and outcome variables, which precluded their possible inclusion in pooled or meta- analysis. The mean NOS and modified Coleman scores of respectively 6 and 52.7 demonstrated an overall poor study quality. Overall, despite our efforts to investigate the effect of some variables, such as age at surgery and surgical technique used, on the outcome, no definite information about potential predictive factors was detected. Finally, we did not demonstrate publication biases due to the potential overrepresentation of the positive outcomes. In order to highlight potential unusual and rare adverse events, we also screened case reports. Nonetheless, we are aware of the possible bias due to the lack of publications of unfavorable results.

## 5. Conclusions

Even considering these shortcomings, our systematic review may help to assess the effectiveness, safety, potential results and complications of surgery for SD. Although we have demonstrated homogeneous satisfactory results and a low rate of complications, regardless of the technique used, the present results should be interpreted with caution as they are derived from studies affected by a number of relevant limitations, potentially biasing the obtained results. Larger and well-designed longitudinal multicentric studies, using patient-derived outcomes, are required to better compare the different modalities of treatment for SD and to improve future clinical practice.

## Figures and Tables

**Table 1:** Grading of the included studies (Newcastle–Ottawa Scale (0–9); Modified Coleman Methodology Score (0–100)).

	No. of Cases	Newcastle–Ottawa Scale	Modified Coleman Methodology Score
Alsiddiky et al. (2020) [17]	23	6	57
Abuhassan et al. (2011) [35]	13	6	52
Agarwal et al. (2018) [36]	8	6	54
Ahmad (2010) [37]	11	6	57
Andrault et al. (2009) [15]	6	6	49
Ashok et al. (2020) [16]	14	8	47
Aslani et al. (2020) [38]	31	6	61
Aydinli et al. (2005) [39]	12	6	51
Bellemans (1999) [40]	7	6	57
Bhasker et al. (2011) [41]	7	8	51
Borges et al. (1996) [42]	16	6	52
Carson (1981) [33]	11	6	43
Chung et al. (1976) [43]	5	6	42
Da Silva Reginaldo et al. (2009) [44]	9	6	46
Dhir et al. (2018) [21]	5	6	50
Di Gennaro et al. (2012) [45]	56	6	61
Elzohairy et al. (2019) [46]	10	6	54
Farsetti et al. (2003) [32]	8	6	44
Gonen et al. (2010) [47]	23	6	57
Greitemann et al. (1993) [23]	37	6	48
Grogan et al. (1983) [2]	13	6	58
Jiang et al. (2019) [48]	34	6	61
Jindal et al. (2012) [49]	12	6	54
Khairouni (2002) [50]	17	2	25
Klisić et al. (1981) [51]	28	2	45
Leibovic (1990) [34]	15	6	60
Masquijo et al. (2009) [19]	14	8	71
McMurtry et al. (2005) [52]	12	6	57
Mears (2001) [18]	8	6	57
Naik et al. (2020) [53]	40	6	61
Nakamura et al. (2016) [54]	14	6	57
Öner et al. (2020) [9]	17	6	55
Patwardhan et al. (2019) [55]	28	6	51
Siu et al. (2011) [56]	8	6	57
Sulamaa et al. (1954) [13]	4	6	44
Vuillermin et al. (2020) [57]	24	6	46
Wada et al. (2014) [14]	22	6	54
Walstra et al. (2013) [58]	7	8	60
Wilkinson (1980) [59]	12	6	54
Yamada et al. (2013) [60]	7	6	54
Zhang et al. (2006) [20]	26	6	58

**Table 2:** Characteristics of patients included in the statistical analysis.

Factor	Level	Value
No. of patients		236
Study	Abuhassan et al. (2011) [35]	13 (5.5%)
	Agarwal et al. (2018) [36]	8 (3.4%)
	Ahmad (2010) [37]	15 (6.4%)
	Alsiddiky et al. (2020) [17]	23 (9.7%)
	Andrault et al. (2009) [15]	6 (2.5%)
	Bhasker et al. (2011) [41]	7 (3.0%)
	Da Silva Reginaldo et al. (2009) [44]	9 (3.8%)
	Dhir et al. (2018) [21]	5 (2.1%)
	Elzohairy et al. (2019) [46]	10 (4.2%)
	Farsetti et al. (2003) [32]	8 (3.4%)
	Masquijo et al. (2009) [19]	14 (5.9%)
	McMurtry et al. (2005) [52]	12 (5.1%)
	Patwardhan et al. (2019) [55]	28 (11.9%)
	Wada et al. (2014) [14]	23 (9.7%)
	Walstra et al. (2013) [58]	8 (3.4%)
	Wilkinson (1980) [59]	12 (5.1%)
	Yamada et al. (2013) [60]	7 (3.0%)
	Zhang et al. (2006) [20]	28 (11.9%)
Period of treatment	<1990	12 (5.1%)
	2000–2009	77 (32.6%)
	≥2010	147 (62.3%)
Age at treatment (mean in years), median (IQR)		6 (4.7)
Surgical technique	Green/Schrock	71 (30.1%)
	Mears	21 (8.9%)
	Osteotomy	32 (13.6%)
	Resection	35 (14.8%)
	Woodward	77 (32.6%)
Cavendish classification	2	31 (14.0%)
	3	137 (62.0%)
	4	53 (24.0%)
Rigault classification	2	41 (43%)
	3	54 (57%)

**Table 3:** Assessment of the Cavendish and Rigault scores according to the surgical technique.

	Grade	Green/Schrock	Woodward	Osteotomy	Resection	Mears
Preoperative Cavendish’s score	2	0 (0%)	14 (18%)	4 (13%)	11 (33%)	2 (10%)
	3	38 (63%)	42 (55%)	27 (84%)	16 (48%)	15 (71%)
	4	22 (37%)	20 (26%)	1 (3%)	6 (18%)	4 (19%)
Postoperative Cavendish’s score	1	31 (52%)	41 (54%)	19 (59%)	14 (42%)	17 (81%)
	2	24 (40%)	33 (43%)	12 (38%)	15 (45%)	4 (19%)
	3	5 (8%)	2 (3%)	1 (3%)	4 (12%)	0 (0%)
Preoperative Rigault’s score	2	6 (18%)	18 (46%)	7 (88%)	5 (71%)	5 (71%)
	3	28 (82%)	21 (54%)	1 (13%)	2 (29%)	2 (29%)
Postoperative Rigault’s score	1	26 (76%)	28 (72%)	1 (13%)	4 (57%)	
	2	8 (24%)	11 (28%)	7 (88%)	2 (29%)	
	3	0 (0%)	0 (0%)	0 (0%)	1 (14%)	

## Supplementary Materials

The following are available online at https://www.mdpi.com/article/10.3390/children8121142/s1, Table S1: Demographic details of the included studies; Table S2: Surgical operative details and complications of the studies.
