# Supplementary material for: Surgical Treatment of Sprengel’s Deformity: A Systematic Review and Meta-Analysis
Source: Children (Basel). 2021 Dec 6;8(12):1142. doi: 10.3390/children8121142 (PMC8700527; doi:10.3390/children8121142)
Supplement: Supplementary file 1 [file children-08-01142-s001.zip › children-1472482-supplementary.pdf]

Supplementary Table S1: Demographic details of the included studies.

	No of cases	Sex M: male F: female	Affected side R: right L: left bil: bilateral	Associated anomalies	Pre operative ROM (elevation/abduction) (grades)	Type of connection: O: osseous C: cartilagineous F: fibrous	Pre operative Cavendish's classification	Pre operative Rigault's classification	Mean age at surgery (years)
Alsiddiky et al. (2020)[32]	23	9 M<br>14 F	9 R<br>14 L	9 KF<br>3 SB<br>2 SC	NA/97.39	6 O<br>2 C<br>2 F	17(3); 6(4)	7(2); 16(3)	6.6
Abuhassan et al. (2011)[45]	13	3 M<br>10 F	4 R<br>9 L	2 KF<br>6 SC<br>3 ribs anomalies<br>3 spinal cord anomalies	NA/110	6 O	5(3); 8(4)	NA	5.9
Agarwal et al. (2018)[29]	8	2 M<br>6 F	3 R<br>5 L	2 KF<br>2 SB<br>5 vertebral anomalies	NA/123.75	2 O	1(2); 7(3)	7(2); 1(3)	6.9
Ahmad (2010)[41]	11	5 M<br>6 F	3 R<br>4 L<br>4 bil	5 KF<br>6 SB<br>4 SC<br>2 unilateral kidneys<br>1 ribs anomaly<br>2 vertebral anomalies<br>1 deafness	NA/ <90	7 O	15(3)	NA	5.6
Andrault et al. (2009)[24]	6	5 M<br>1 F	6 L	2 SC<br>1 pectus carinatum	143.33/106.66	4 O	NA	1(2); 5(3)	3.8

Ashok et al. (2020)[36]	14	3 M<br>11 F	4 R<br>9 L<br>1 bil	6 KF<br>4 SC	116/108	10 O	9(3); 6(4)	8(2); 7(3)	4.5
Aslani et al. (2020)[52]	31	9 M<br>22 F	18 R<br>9 L<br>4 bil	14 SC	132/123	7 O	2.9	NA	7.3
Aydinli et al. (2005)[21]	12	2 M<br>10 F	4 R<br>8 L	3 KF<br>3 SC	95/81	5 O	NA	NA	5.2
Bellemans (1999)[42]	7	4 M<br>3 F	3 R<br>4 L	1 KF<br>1 Goldenhar syndrome<br>1 anovaginal fistula	NA/88.57	NA	NA	NA	6.1
Bhasker et al. (2011)[27]	7	3 M<br>4 F	3 R<br>4 L	2 KF<br>1 unilateral kidney	84.28/95.71	6 O	2(2); 5(3)	5(2); 2(3)	4.3
Borges et al. (1996)[39]	16	2 M<br>14 F	10 R<br>5 L<br>1 bil	7 KF<br>8 SC<br>9 spinal and ribs anomalies<br>2 kidney anomalies<br>1 finger anomaly<br>1 gastroenteric anomaly	NA/115	4 O	2(2); 12(3); 1(4)	NA	8.3
Carson (1981)[38]	11	3 M<br>8 F	6 R<br>3 L<br>2 bil	2 KF<br>3 SB<br>7 SC<br>2 unilateral kidneys<br>6 rib anomalies<br>1 situs inversus	NA/127.69	2 O	The majority were 2 and 3	NA	8.4

				1 finger deformity<br>1 absent pectoralis major muscle<br>1 hypoplastic ear					
Chung et al. (1976)[40]	5	NA	4 unilateral<br>1 bil	4 KF<br>2 renal hypoplasia<br>2 cervical ribs	NA/NA	4 O<br>1 F	NA	NA	5.8
Da Silva Reginaldo et al. (2009)[43]	9	7 M<br>2 F	1 R<br>8 L	4 KF<br>3 SC<br>3 ribs anomalies<br>2 renal anomalies<br>1 vertebral anomaly<br>1 cleft palate<br>1 umbilical hernia	110/NA	1 O	5(3); 4(4)	NA	6.9
Dhir et al. (2018)[16]	5	3 M<br>2 F	NA	5 craniocervical anomalies<br>2 unilateral kidneys	116/110	5 O	5(4)	4(2); 1(3)	NA
Di Gennaro et al. (2012)[31]	56	25 M<br>31 F	20 R<br>31 L<br>5 bil	25 KF<br>27 SC<br>11 chest asymmetries<br>2 unilateral kidneys<br>5 facial asymmetries<br>2 cleft palates	121/92	22 O<br>6 F<br>3 C	41(3); 20(4)	41(2); 20(3)	6.4

				2 finger anomalies					
Elzohairy et al. (2019)[44]	10	2 M<br>8 F	4 R<br>6 L	NA	83/NA	5 O	5(3); 5(4)	NA	2.8
Farsetti et al. (2003)[20]	8	1 M<br>7 F	4 R<br>4 L	4 KF<br>2 SB	NA/103.12	4 O	NA	2(2); 6(3)	8
Gonen et al. (2010)[14]	23	16 M<br>7 F	19 unilateral<br>4 bil	2 KF<br>3 SC<br>1 unilateral kidney	NA/101.3	13 O	3.4	NA	4.5
Greitemann et al. (1993)[17]	37	27 M<br>10 F	NA	18 SC<br>30 rib anomalies	123/128	7 O	10(1); 9(2); 15(3); 3(4)	NA	8
Grogan et al. (1983)[2]	13	3 M<br>10 F	7 R<br>5 L<br>1 bil	9 KF<br>8 SC<br>8 rib and vertebral anomalies<br>6 spinal cord anomalies<br>3 cleft palate<br>2 congenital hip dislocations<br>1 unilateral kidney<br>1 congenital heart disease<br>1 deafness<br>1 finger anomaly<br>1 accessory nipple<br>1 bifid clavicle	NA/113.57	13 O	3.4	NA	6.9

Jiang et al. (2019)[30]	34	18 M<br>16 F	22 R<br>14 L<br>2 bil	6 KF<br>7 SC<br>4 SB<br>3 rib anomalies<br>2 torticollis<br>1 foot anomaly	NA/102.3	20 O	22(3); 12(4)	23(2); 11(3)	5.6
Jindal et al. (2012)[53]	12	5 M<br>7 F	6 R<br>6 L	2 SC<br>1 rib anomaly<br>1 foot anomaly	NA/115.8	4 O	3.2	NA	5.6
Khairouni (2002)[13]	17	5 M<br>12 F	13 unilateral<br>4 bil	2 KF<br>3 SB<br>2 SC, 1 kyphosis<br>1 rib anomaly<br>2 vertebral anomalies<br>5 hypoplastic fibrous muscles	NA/84.3	11 O	NA	NA	NA
Klisić et al. (1981)[26]	28	9 M<br>19 F	19 R<br>8 L<br>1 bil	1 KF<br>12 SC<br>11 rib anomalies<br>2 vertebral anomalies<br>1 multiple finger anomaly<br>1 pterigium colli	NA/NA	4 O	NA	NA	NA
Leibovic (1990)[10]	15	NA	2 R<br>11 L<br>2 bil	2 KF<br>5 SC<br>2 vertebral anomalies<br>3 unilateral kidneys<br>1 pelvic kidney<br>1 undescended testes	NA/91	6 O	NA	NA	3.9

Masquijo et al. (2009)[33]	14	5 M<br>9 F	6 R<br>8 L	3 KF<br>5 SC<br>1 spinal anomaly<br>2 rib anomalies<br>2 foot and finger anomalies	83.92/81.07	11 O	10(3); 4(4)	9(2); 5(3)	6.3
McMurtry et al. (2005)[48]	12	4 M<br>8 F	8 R<br>4 L	3 KF<br>2 SC<br>2 SB<br>2 vertebral anomalies<br>2 Erb's palsy<br>2 hydrocephalus<br>1 finger anomaly	NA/90	7 O	1(2); 10(3); 1(4)	NA	8.1
Mears (2001)[25]	8	5 M<br>3 F	4 R<br>4 L	2 KF	100/90.62	2 O	NA	NA	5.7
Naik et al. (2020)[35]	40	15 M<br>25 F	22 R<br>18 L	4 KF<br>20 SC<br>13 rib anomalies<br>1 unilateral kidney<br>2 congenital heart diseases<br>2 spinal cord anomalies	NA/NA	8 O	6(2); 34(3)	35(2); 5(3)	5
Nakamura et al. (2016)[18]	14	8 M<br>6 F	NA	7 KF<br>6 SC<br>1 SB<br>1 rib anomaly	100/100	7 O	NA	NA	4.4

				1 unilateral kidney<br>1 accessory auricle<br>1 cleft palate<br>1 bladder exstrophy					
Oner et al. (2020)[49]	17	5 M<br>12 F	9 R<br>7 L<br>1 bil	3 KF	96.4/93.1	NA	3(2);12(3);3(4)	NA	8.5
Patwardhan et al. (2019)[50]	28	9 M<br>19 F	12 R<br>16 L	2 KF	98/108.21	8 O	14(2); 8(3); 6(4)	NA	6.3
Siu et al. (2011)[22]	8	3 M<br>5 F	2 R<br>6 L	2 SC<br>4 vertebral anomalies<br>1 congenital torticollis<br>1 hydrocephalus<br>1 finger anomaly	NA/122.14	7 O<br>1 F	NA	NA	6.3
Sulamaa et al. (1954)[37]	4	3 M<br>1 F	2 R<br>2L	2 KF<br>2 SC<br>1 SB<br>2 rib anomalies<br>1 spinal cord anomaly<br>1 vertebral anomaly	NA/120	1 O	NA	NA	4
Vuillermin et al. (2020)[11]	24	NA	NA	Not reported	NA/90	15 O	NA	10(2); 14(3)	4.8
Wada et al. (2014)[28]	22	13 M<br>9 F	5 R<br>16 L	19 KF<br>3 SC	NA/88.26	1 O<br>6 F	13(3); 10(4)	4(2); 19(3)	2.9

			1 bil	21 SB<br>2 radial ray deficiencies		13 C			
Walstra et al. (2013)[9]	7	5 M<br>2 F	3 R<br>3 L<br>1 bil	2 KF<br>3 SC<br>1 SB<br>1 rib anomaly<br>1 congenital heart disease	NA/112.5	3 O	8(3)	8(2)	8.8
Wilkinson (1980)[15]	12	3 M<br>9 F	NA	1 KF<br>5 SC, 1 hyperkyphosis<br>2 SB<br>2 vertebral anomalies<br>4 rib anomalies<br>1 unilateral kidney<br>1 facial asymmetry	NA/117.91	4 O	2(2); 10(3)	NA	7.7
Yamada et al. (2013)[34]	7	2 M<br>5 F	2 R<br>5 L	4 KF<br>1 SC<br>3 SB<br>1 Waardenburg syndrome	97.14/99.28	5 O<br>1 F	4(3); 3(4)	3(2); 4(3)	4.1
Zhang et al. (2006)[51]	26	15 M<br>11 F	6 R<br>18 L<br>2 bil	1 KF<br>1 vertebral anomaly	NA/111.07	11 O<br>5 F<br>1 C	11(2); 16(3); 1(4)	NA	5.7

Supplementary Table S2: Surgical operative details and complications of the studies.

	No of cases	Operative procedure	Follow up (years)	Immobilisation time (weeks)	Postoperative ROM (elevation/abduction) (grades)	Post operative Cavendish's classification	Post operative Rigault's classification	Complications	Clavien Dindo Sink classification (1, 2, 3,4, 5)
Alsiddiky et al. (2020)[32]	23	Modified Woodward	5.5	2	NA/149.13	8(1); 14(2); 1(3)	16(1); 7(2)	No	0
Abuhassan et al. (2011)[45]	13	Modified Green	5.9	4	NA/166.53	10(1); 3(2)	NA	Scar's widening<br>Ossification<br>Seroma	1<br>2<br>3
Agarwal et al. (2018)[29]	8	Vertical Scapular Osteotomy	4.2	1	NA/145	4(1); 4(2)	1(1); 7(2)	No	0
Ahmad (2010)[41]	11	Green	3	NA	NA/138.53	7(1); 8(2)	NA	Winging of the scapula (4)<br>Keloid (4)	2<br>1
Andrault et al. (2009)[24]	6	Modified Green	4.5	1	165/159.16	NA	4(1); 2(2)	Hypertrophic scar<br>Transient brachial plexus palsy	1<br>2
Ashok et al. (2020)[36]	14	Woodward	4.6	NA	155.33/145.33	8(1); 7(2)	11(1); 4(2)	Suture granuloma<br>Hypertophic scar (2)	3<br>1
Aslani et al. (2020)[52]	31	Vertical Scapular Osteotomy	7.3	2	163/159	1.7	NA	Hypertrophic scar (3)<br>Superficial infection	1<br>2

Aydinli et al. (2005)[21]	12	Modified Green	2	5	105/97	NA	NA	Unsightly surgical scar (3)	1
Bellemans (1999)[42]	7	Modified Green	5	3	NA/170	NA	NA	Keloid	1
Bhasker et al. (2011)[27]	7	Mears	2.1	2	135.71/147.14	7(1)	NA	Keloid (2)	1
Borges et al. (1996)[39]	16	Woodward	8	NA	NA/150	8(1); 6(2); 1(3)	NA	Unsightly scar (2)<br>Winging of the scapula (2)<br>Transient brachial plexus palsy	1<br>2<br>2
Carson (1981)[38]	11	Woodward	6.2	NA	NA/156.92	6(1); 2(2)	NA	Unsightly scarring (7)	1
Chung et al. (1976)[40]	5	Woodward	4.5	NA	NA/NA	NA	NA	No	0
Da Silva Reginaldo et al. (2009)[43]	9	Modified Green	3.6	4	148.88/NA	6(1); 3(2)	NA	Superficial infection<br>Broken wire (3)	2<br>3
Dhir et al. (2018)[16]	5	Omovertebral bar resection	2	NA	158/158	3(1); 2(2)	4(1); 1(2)	No	0
Di Gennaro et al. (2012)[31]	56	Modified Green	10.9	2	155/112	22(2); 32(3);7(4)	15(1); 34(2); 12(3)	Hypertrophic scar (8)<br>Keloid (16)<br>Incomplete transient brachial palsy (3)<br>Recurrence of the bony bar	1<br>1<br>2<br>3

Elzohairy et al. (2019)[44]	10	Woodward	3.7	2	152.5/NA	6(1); 4(2)	NA	Superficial infection	2
Farsetti et al. (2003)[20]	8	Woodward<br>Putti-Schrock's<br>Partial scapular resection	28	2	NA/141.25	NA	7(2); 1(3)	Unsightly surgical scar (4)<br>Regeneration of the upper part of the scapula	1<br>3
Gonen et al. (2010)[14]	23	Modified Green	11.3	4	NA/145.93	1.9	NA	Skin necrosis<br>Hypertrophic scars (6)<br>Winging of the scapula (2)	2<br>1<br>2
Greitemann et al. (1993)[17]	37	König-Wittek<br>Green<br>Woodward<br>Partial scapular resection	10	NA	NA/147	1.6	NA	Keloid (4)<br>Osteophytes (3)<br>Transient brachial plexus palsy	1<br>2<br>2
Grogan et al. (1983)[2]	13	Woodward	2.5	2	NA/147.85	7(1); 5(2); 1(3); 1(4)	NA	Winging of the scapula<br>Transient brachial plexus palsy	2<br>2
Jiang et al. (2019)[30]	34	Modified Green	6.1	2-3	NA/142.6	8(1); 24(2); 2(3)	30(1);4(2)	Hypertrophic scar or keloid (8)	1
Jindal et al. (2012)[53]	12	Woodward	2.6	3	NA/153.3	1.25	NA	No	0
Khairouni (2002)[13]	17	Modified Woodward	NA	6	NA/107.9	NA	NA	Superficial infections (2)	2

Klisić et al. (1981)[26]	28	Green	NA	3	NA/NA	NA	NA	Deep infections (2)<br><br>Opening of the pleura	2<br>3(one patient required a second surgery)<br><br>3
Leibovic (1990)[10]	15	Modified Green	6.6	4	NA/148	NA	NA	Unsightly surgical scar (2)<br>Mild winging of the scapula	1<br><br>1
Masquijo et al. (2009)[33]	14	Mears	3.7	1	152.14/145	10(1); 4(2)	NA	Hypertrophic scar (4)<br>Ossifications (2)<br>Keloid (2)	1<br>3<br>1
McMurtry et al. (2005)[48]	12	Vertical Scapular Osteotomy	9.6	1	NA/130.76	7(1); 4(2); 1(3)	NA	Recurrence of upper plexus palsy	2
Mears (2001)[25]	8	Mears	5.5	2 days	175/159.28	NA	NA	Keloid (2)<br>Ossification	1<br>3
Naik et al. (2020)[35]	40	Modified Green	5.2	6	NA/NA	0.25	1.1	Winging of the scapula (6)<br>Brachial plexus palsy and lost pulsation<br>Skin necrosis (2)	2<br><br>3<br><br>2<br>3 (one patient required a

								Pleural tear	secondary closure)<br>3
Nakamura et al. (2016)[18]	14	Woodward	8.8	2	160/160	NA	NA	No	0
Oner et al. (2020)[49]	17	Woodward	5.2	3	137.5/149.2	2(0);6(1);10(2)	NA	Scar's widening (4)	1
Patwardhan et al. (2019)[50]	28	Modified Woodward	1.7	3	108.6/142.5	18(1); 10(2)	NA	Superficial infection	2
Siu et al. (2011)[22]	8	Woodward	9.4	1	NA/157	NA	NA	No	0
Sulamaa et al. (1954)[37]	4	Putti-Schrock's	1	6	NA/180	NA	NA	Broken wire<br>Partial recurrence	2<br>2
Vuillermin et al. (2020)[11]	24	Woodward	3.4	NA	NA/110	NA	5(1);14(2);2(3)	Reformation of excised omovertebral bone and stiffness (2)<br>Fracture of clavicle at the previous morselization site	2<br>3 (only one patient was reoperated)<br>2
Wada et al. (2014)[28]	22	Modified Green	4.4	2-4	NA/150.86	8(1); 10(2); 5(3)	22(1); 1(2)	Unsightly scarring (8)<br>Winging of the scapula (3)<br>Transient brachial plexus palsy (2)	1<br>2<br>2

Walstra et al. (2013)[9]	7	Woodward	13.5	8	NA/170	6(1); 2(2)	8(1)	Pleural lesion<br>Unsightly scarring (5)<br>Wound dehiscence (2)	3<br>1<br>1
Wilkinson (1980)[15]	12	Vertical Scapular Osteotomy	10	6	NA/163.75	1 (0); 7(1); 4(2)	NA	Transient brachial plexus palsy<br>Little prominence of the inferior angle	2<br>1
Yamada et al. (2013)[34]	7	Woodward	4.4	1	160/161.42	3(1); 3(2); 1(3)	4(1); 3(2)	Keloid	1
Zhang et al. (2006)[51]	26	Partial scapular resection	3.8	2	NA/151.42	11(1); 13(2); 4(3)	NA	Winging of the scapula	1
